# Neuromagnetic brain responses to words from semantic sub- and supercategories

**DOI:** 10.1186/1471-2202-6-57

**Published:** 2005-08-31

**Authors:** Ramin Assadollahi, Brigitte Rockstroh

**Affiliations:** 1 Department of Psychology, University of Konstanz, P.O. Box D23 D-78457 Konstanz, Germany

## Abstract

**Background:**

We explored spatio-temporal patterns of cortical activity evoked by written words from super-ordinate and sub-ordinate semantic categories and hoped to find a differential cortical and/or temporal distribution of the brain response depending on the level of the categories. Twenty-three subjects saw 360 words belonging to six sub-ordinate categories (mammals, birds, fish, fruit, flowers, trees) within two super-ordinate categories (fauna, flora). Visually evoked magnetic fields were determined from whole-head (148-sensor) magnetoencephalography and analyzed in the source space (Minimum Norm Estimate).

**Results:**

Activity (MNE amplitudes) 100–150 ms after stimulus onset in the left occipito-temporal area distinguished super-ordinate categories, while later activity (300–550 ms) in the left temporal area distinguished the six sub-ordinate categories.

**Conclusion:**

Our results document temporally and spatially distinct processing and representation of words according to their categorical information. If further studies can rule out possible confounds then our results may help constructing a theory about the internal structure of entries in the mental lexicon and its access.

## Background

The mental lexicon is usually considered to be a compilation of linguistic information, with each word having an entry. The precise structure of a lexical entry continues to be a topic of investigation and discussion. From a linguistic perspective, a lexical entry for a word should contain information about its pronunciation, its orthography, its syntactic properties, and its semantic information. In the present study, we explored the aspect of categories within semantic information. A sparrow for example, might belong to the category *bird *as well as to the category *animal*. How and where is such information encoded in the brain? Is the category information represented within one brain compartment or in a distributed fashion?

These questions are of course not only restricted to the mental lexicon but also apply to the field of visual object recognition, for example. Cognitive neuroscience has contributed to these questions by considering evidence on the representation of object categories in the brain (mainly derived from picture naming tasks): For visually presented exemplars of particular object categories the use of functional brain imaging disclosed activity in the ventral pathway of the visual system, with activity distinguishing different object categories (faces, animals, fruits, buildings, chairs, and man made tools) along the occipito-temporal pathway, which was thereupon labelled 'what-stream' (e.g., [1-9]).

Evidence from brain responses distinguishing between sub- and super-ordinate category information is still limited. For instance, Löw and colleagues [10] examined the spatial distribution of sources of neuromagnetic activity elicited by pictures of super- (e.g. animal, furniture etc) and sub-ordinate (e.g., deer, wolf; table, chair, etc.) object categories. Significantly higher similarity (correlation) of evoked magnetic responses within than across super-ordinate categories indicated macroscopically distinct representation of defined semantic categories. Activation of these representations was most prominent in the right parietal cortex around 200 ms and in the left parieto-temporal cortex around 400 ms after stimulus onset. This spatio-temporal sequence supports sequential perceptual dynamics of the categorization process, with initial activation of representation in the extrastriate areas of the right hemisphere followed by activity along the ventral processing stream primarily in the left temporal lobe.

This evidence served as the background for the present study, which aimed at clarifying the dynamics involved in the lexical access of words. Representation and activation of semantic categories were probed by analyzing spatio-temporal brain activity that was induced by written words. They a) were related to different super- and sub-ordinate categories; b) came from the natural domain (in order to rule out/control for possible effects of gross features of the man-made/nature-made difference); c) were of low to ultra-low frequency (exploiting evidence that low-frequent words induce marked event-related brain responses, [11]). Magnetic source imaging (MSI, the analysis of sources of activation from high-resolution magnetoencephalography) was used as an indication of cortical representation. In particular, we aimed at clarifying the following questions: a) whether visually presented words related to different super- and sub-ordinate natural categories would be represented in spatially separable areas along the occipital-to-temporal visual pathway, corresponding to the 'what stream' determined for visually presented object categories; b) whether distinct activity patterns primarily in the left hemisphere would indicate the representation of (additional) linguistic features for semantic categories activated by words; c) whether spatio-temporal brain responses distinguish super- and sub-ordinate categories as an indication of feature-directed category representation and access of lexical entries.

## Results and discussion

**Figure 1:**
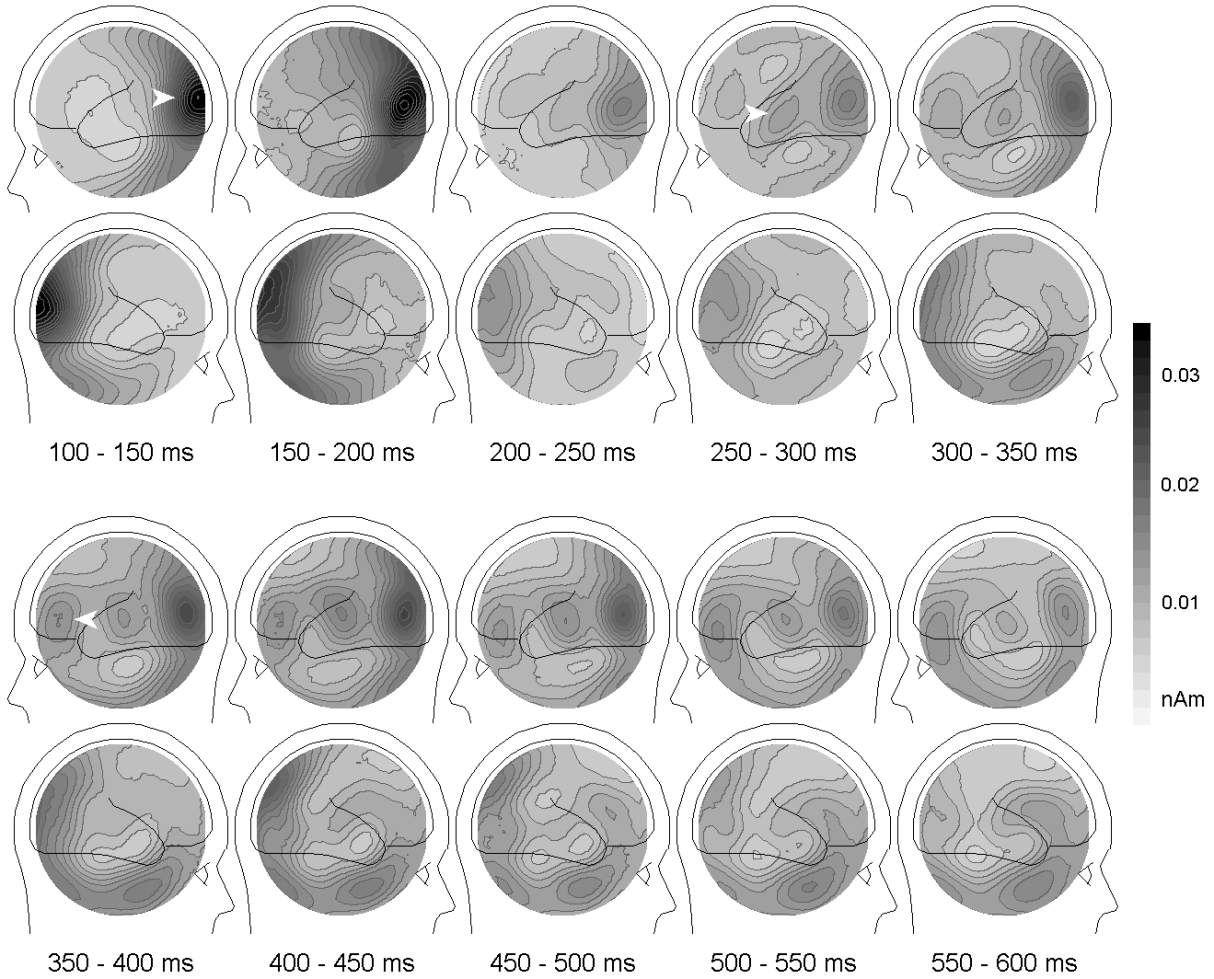
**Topographies**. Topographical distribution of source activity (MNE amplitudes) across 50-ms intervals in the left (upper rows) and right (lower rows) hemisphere, averaged across all stimuli and subjects. Darker areas indicate stronger source activation (MNE-amplitudes).

Highly similar activity patterns were elicited by all six sub-ordinate categories and we did not observe gross topographical differences in response to different categories. Therefore, Figure 1 illustrates the course of activity analyzed in the source space and averaged across the six categories. A left occipital focus of activity is prominent already for the first interval 100–150 ms after stimulus-onset and remains obvious throughout the recording period; starting around 250 and 300 ms, an additional left-temporal focus becomes evident (see arrow in Fig. 1); as a third prominent focus, left-anterior activation starting around 350 ms can be taken form Fig. 1.

As is also obvious from Figure 1, activity was prominent in the left hemisphere and negligible in the right hemisphere. Since effects of stimulus categories on activity patterns were statistically confirmed only for the left but not for the right hemisphere, the subsequent results refer to the left-hemisphere foci.

**Figure 2:**
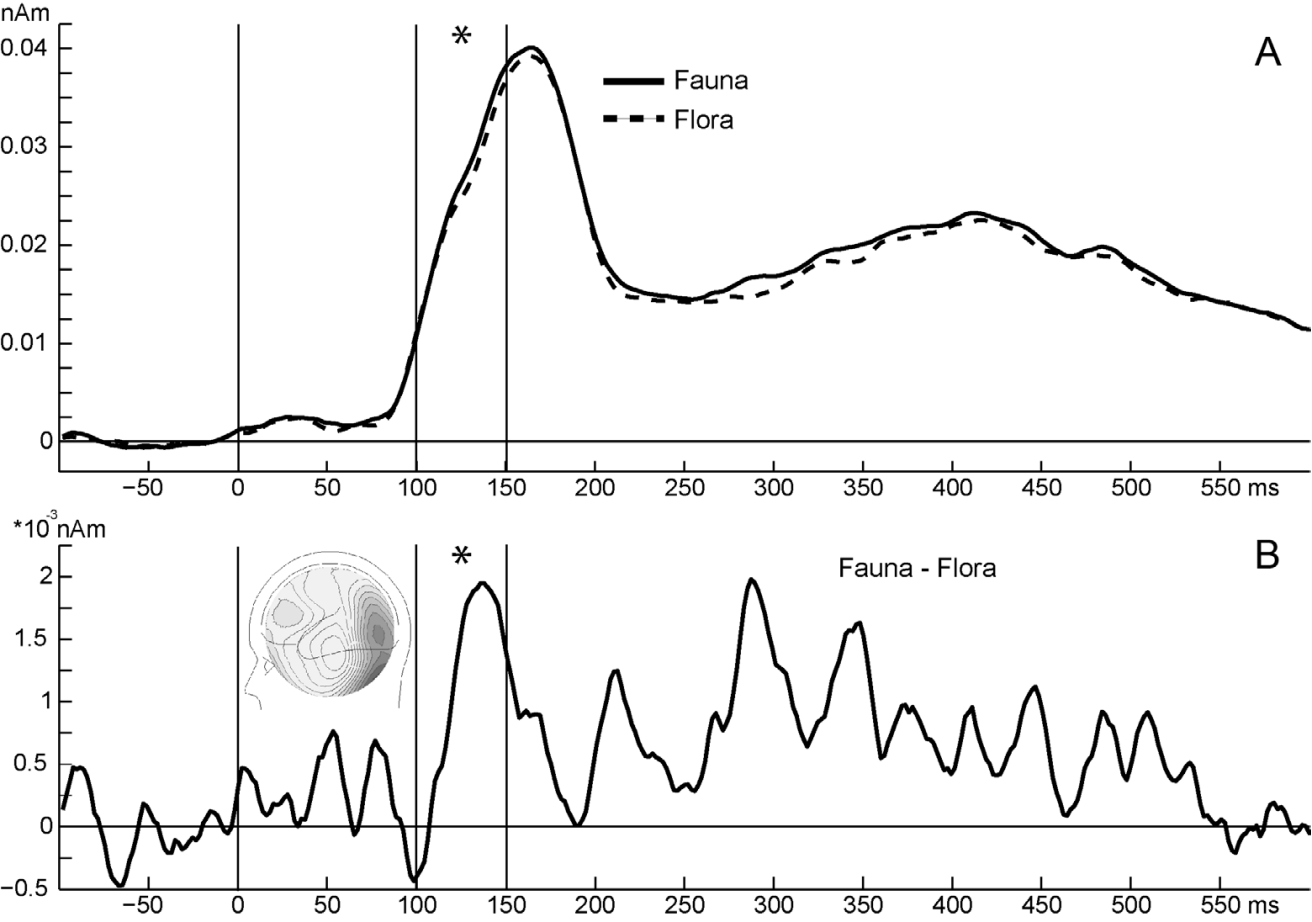
**Source waveforms and difference waves for left occipital area**. (a) Source waveform for left occipital area. Time course of activity (amplitudes of MNE in nAm) in the left-occipital area across 100 ms pre- and 600 ms post-stimulus onset averaged across subjects separately for the super-ordinate categories (fauna: bold, flora: dashed). (b) Difference waves for left occipital area. Time course of the difference in brain activity (amplitudes of MNE in nAm in the left-occipital area) between super-ordinate categories (fauna – flora). The time interval with the significant difference is marked by asterisks, an illustrative difference map is given as insert.

Figure 2a illustrates the time course of source activity (amplitudes of Minimum Norm Estimates) at the left occipital area (covering 7 dipoles) separately for the two super-ordinate categories. More pronounced activity induced by fauna- compared to flora-items is indicated around 150 ms after stimulus-onset, which is confirmed for the time segment in the difference curve (Figure 2b) and by a main effect SUPERCATEGORY (comparing the super-ordinate categories; F(1,22) = 4.7, p < .05). Differences in subsequent time segments fell short of significance. The source localization of this effect may suggest that this activity is related to processing of the visual word form in the so-called Visual Word Form Area (VWFA, c.f. Salmelin group: [12], Dehaene group: [13]). The comparison with results from our own study [14] using the same localization methodology as in the present study revealed that the two areas are in fact distinct. The focus of activity appears to be more anterior and more superior compared to the focus reported in [14]. This suggests that the activation difference in the SUPERCATEGORY is not due to simple visual word form processing (and possibly related factors like number of vowels, morphemes, syllables, word frequency and the like). Studies finding differences in the VWFA, usually modulated low level properties of the stimulus material such as words vs. non-words vs. pseudo-words vs. symbols. Our own study [14] manipulated word length and frequency. Whereas the modulation of length revealed different amplitudes in the occipital lobe, the differential activation due to variation of word frequency was localized at the VWFA. We are not aware of any study that found different activations due to semantic variation of the stimulus material. Thus, effects of wordness and semantics may occur in similar time ranges but at different areas in the brain.

**Figure 3:**
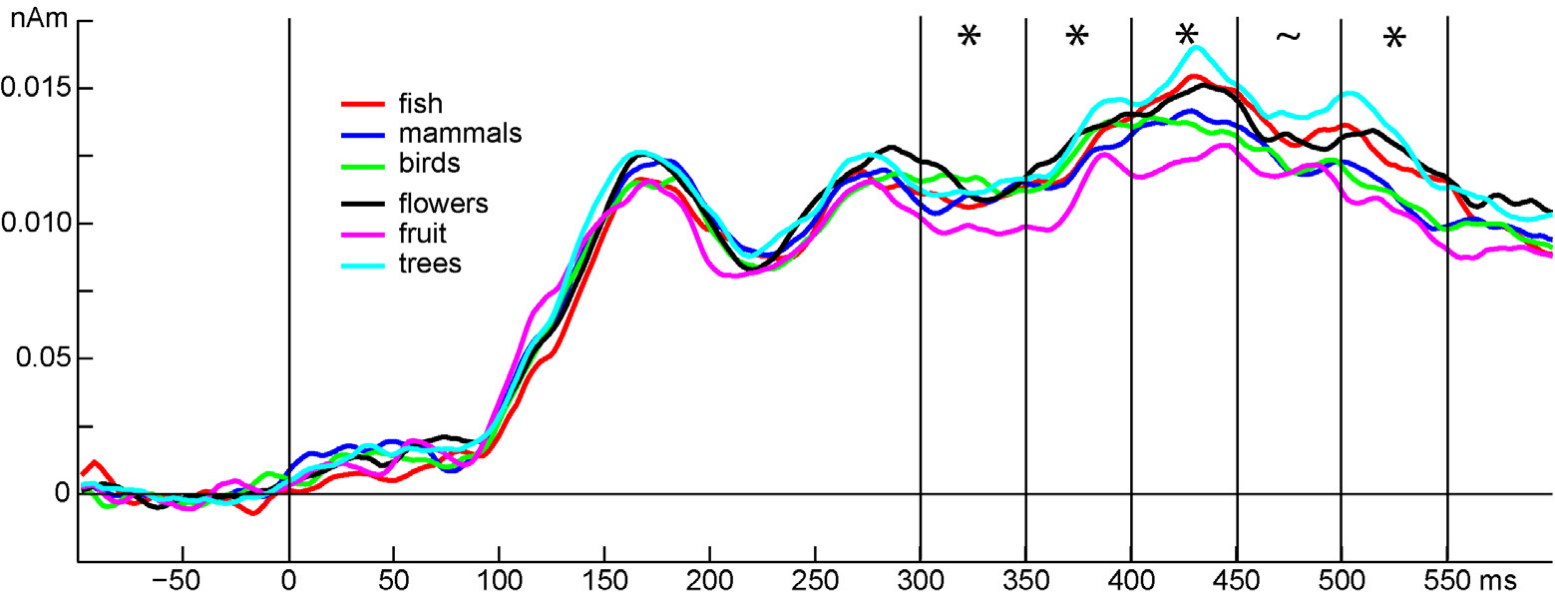
**Source waveforms for left temporal area**. Time course of brain activity (amplitudes of MNE in nAm) for 100 ms pre- and 600 ms post-stimulus onset averaged across subjects separately for the six sub-ordinate categories in the left-temporal area. Significant interactions are marked by asterisks, the marginally significant interaction by a tilde.

The time course of activity for the left temporal region (Figure 3) discloses differences between sub-ordinate categories between 300 and 550 ms after stimulus onset. Interactions SUPER × SUBCATEGORY indicate significant differences between sub- and super-ordinate categories for the intervals 300–350 ms (F(2,44) = 3.3, p < .05), 350–400 ms (F(2,44) = 3.1; p = .05), and 400–450 ms (F(2,44) = 3.8, p < .05). For the last interval post-hoc tests verified significant differences between the sub-categories fruit vs flowers (p < .05), fruit vs. trees (p < .01) and fruit vs. fish (p < .01) and marginally significant trends (p < .1) for differences between birds vs fruit, birds vs trees, and mammals vs fruit. Distinct category processing remained apparent until 550 ms after stimulus onset, with the difference for 450–500 ms approaching significance (p < .1) and the difference between 500 and 550 ms (F(2,44) = 4.0; p < .05) being explained by significant post-hoc comparisons of fruit vs. flowers (p < .01), fruit vs. trees (p < .01) and fruit vs. fish (p < .05), flowers vs birds (p < .05) and flowers vs mammals (p < .05), trees vs birds (p < .01) and trees vs mammals (p < .01), and fish vs mammals (p = .05).

In sum, the activity near or at the left temporal pole differentiates between sub-categories. The sub-categories were selected to be semantic in nature. It is not clear whether the activity found resembles an N400 (a component generally reflecting the ease at which a word is integrated into a sentential context), which was also found to be elicited after single words [15]. Also, source localisations of the N400 component do not give a clear picture where the N400 has its cortical origin.

For the left-anterior activity focus indicated in Figure 1, no significant effects of categories were found.

In sum, source analyses of neuromagnetic brain responses to words from different semantic categories, comprising super-ordinate (fauna, flora) and sub-ordinate categorization (mammals, birds, fish; fruit, flowers, trees) disclose specific spatio-temporal activation and a distinction in time and space for sub- versus super-ordinate category features: Whereas super-ordinate features induced different activation already around 100–150 ms after stimulus onset in left-occipital areas, the processing of sub-ordinate category features led to differential activation only later, between 300 and 550 ms, in left-temporal areas. This is, to our knowledge, the first evidence of spatio-temporal separation of category-feature processing in the brain.

### Topography

"Hebbian-type cell assemblies" are described as distributed networks of neurons that are functionally connected by reciprocal dynamic connections and whose strength is modulated based on correlation learning [16,17]. In Pulvermüller's framework of the mental lexicon (see [16] for an overview), the neuronal representation of a word may comprise neurons in the perisylvian cortex (where the acoustic word form is represented) and neurons in areas that process information about object features or associated actions. For nouns denominating objects these features comprise visual associations and, thus, the neuronal network activated by the noun will comprise neurons in the visual association areas in the occipital and inferior temporal lobe. During language acquisition, representations of objects are related to auditory or visual word forms. A cell assembly is established whenever a concept is activated repeatedly and simultaneously with the word form. This cell assembly will then comprise features that reflect parts of its word form representation and parts of the auditory or visual representation of the object the word *refers *to (the so-called *referential *information). From this point of view it makes sense that semantic features of object representations are also shared by the corresponding entries in the mental lexicon. Thus, the same sub-assemblies (reflecting a certain feature) are activated either by the word or by the object itself.

Pulvermüller's work also relates to the research field of the ventral "what-stream". Ishai [18] pointed out that the topological arrangement of visual features in the ventral "what"-stream remains to be clarified. If, as Pulvermüller argues, category-features activated by words are part of category-features activated by corresponding objects, then the present results contribute to this clarification, in that features related to sub-ordinate concepts are processed in areas more anterior to features related to super-ordinate concepts. Gauthier and colleagues [19] pointed out that in visual object recognition the super-ordinate-level (e.g. 'bird'), at which familiar objects are first recognized, was distinguished from more sub-ordinate levels (e.g. 'sparrow'), which required additional perceptual processing. This additional processing related to the sub-ordinate level representation was associated with activation in the fusiform and inferior temporal gyri and the temporal poles. This is in line with the more anterior focus of activity distinguishing sub-ordinate categories in the present study.

In the present study, it is conceivable that the categories fauna and flora activated features related to the visual representations of the respective objects. These features would be part of the representations of the physical, visually perceivable objects and as such part of the ventral "what"-stream of the left occipito-temporal cortex. There are several studies that showed early activation due to referential semantic information (e.g. [20-23]).

While these considerations are supported by the present results of category-specific activity patterns along the left occipito-temporal axis, they should be validated by a direct comparison of cortical activity evoked by pictures and their corresponding words. Chao and colleagues [5] report an fMRI study, where in fact there were similar activations for pictures and their corresponding names.

Why should the representations of sub- and supercategory features be processed at different sites in the brain? There is ample evidence that during the individual development of the brain, maximum neural plasticity starts at primary sensory (e.g. occipital) and motor sites and over time progresses towards secondary areas, parietal areas and finally towards frontal areas [24-28]. Using computer simulations, Shrager and Johnson [29] showed that simple representations of input data can be acquired in early stages of development (i.e. plasticity at primary sites) whereas more abstract representations *can only *be acquired in sites that had high plasticity later on (i.e. more anterior sites). These sites would use the information from the lower level representations together with the input to represent more abstract and/or invariant aspects of the stimulus. In the present case, this would mean that gross categories can be represented early on (in more primary, posterior sites) whereas more fine grained sub-representations will only be acquired later on (in more anterior sites).

### Amplitude

We can only speculate how the amplitude difference in the present study relates to different categories or their features. Generally, there are two reasons for a stronger amplitude in the MEG. Either there are slight topographic differences or the size of the neuronal network activated varies over conditions. Cortical sources may be located on a part of a gyrus where the tangential part of the magnetic field is more *or *less prevalent than in a different condition. The magnetometers used in our 4D Neuroimaging system only capture tangential fields and thus a stronger component in the radial field would mean a reduction in the field strength measured. Thus, the amplitude difference may arise from a slight topographic shift. The other possible answer is that different features have different complexities in their representation. This may translate in different sizes of neuronal networks representing them. The activation of these networks will lead to greater amplitudes for larger networks. Another reason for different network sizes may be the relation of the word to other words. If all words are represented in networks, then these networks may have overlapping parts to reflect the relatedness of words. We tried to capture the associations that subjects had after reading our stimuli and found no systematic variation in the number of associations over categories. Thus, if networks of words (i.e. networks of networks) lead to different amplitudes due to a different degree of connectedness, then this is not a good explanation for the amplitude differences found in the present study. We therefore favour the interpretation of slightly different topographies resulting in different amplitudes.

### Timeline

The present study not only revealed that category related information is distributed *spatially *over the cortex, but it is also activated *over time*. The temporal sequence of cortical activation distinguished the processing super-ordinate (earlier) and sub-ordinate features (later). Considering the results of Gauthier and colleagues [19], we may conclude that features of lexical entries are hierarchically processed, with steps of feature analyses (super-ordinate first) spreading across hundreds of ms.

## Conclusion

The present results may help to speculate over the inner structure of entries in the mental lexicon: Super- and sub-ordinate category features are parts of a word's representation; they activate distinct cortical areas in the left occipital-temporal axis at distinct points in time. Lexical entries may thus be understood as structure of features, represented in distributed networks that are activated by words in a sequential, cascaded way.

## Methods

### Subjects

Twenty-three healthy, right-handed, native German speaking student subjects (12 m, 11f, mean age: 26) volunteers with normal or corrected-to-normal vision were paid (15€ for the two-hour session) for participation in the study.

### Materials & procedure

#### Stimulus selection

Stimuli comprised 360 words related to the two super-ordinate categories (labelled 'fauna' and 'flora') and 6 sub-ordinate categories (fauna: mammals, birds, fish; flora: fruit, flowers, trees). For each sub-ordinate category, 60 stimuli were selected from the CELEX-database [30] for low-frequency (mean frequency was seven per million) and comparable length (8 letters). Since CELEX did not provide enough low frequent nature-made object names meeting these criteria (match for length, frequency < 10), 10 words with ultra-low frequency were added from other sources to each sub-category. We wanted to have as many stimuli as possible to have a broad base of individual concepts sharing their sub- and supercategory features. A better match for other word-level features such as bigram-letter-frequency etc would have further reduced the number of stimuli per category and would thus have reduced the representativeness of the study. The 360 words were assigned to 2 runs, each of 6 series of 30 stimuli (per sub-ordinate category). Within each series the 30 stimuli were presented in pseudo-random order.

#### Behavioural study

Using a computerized test, we presented all 360 stimuli to 48 subjects (30 randomly selected items per subject; subjects did not take part in the MEG study) and asked them to write up their associations. Subjects had 60 seconds time to type in their association for each stimulus. The procedure resulted in four (= 360/48) sets of associations per stimulus, 240 (= 60 × 4) sets of associations per sub-ordinate category and 720 (= 240 × 3) sets of associations per super-ordinate category. We investigated the number of associations per word (i.e. how many associations were made on the average for this particular word) using a computer program and were surprised that this number was surprisingly similar for the super-ordinate categories (fauna: mean = 7.217, SD = 7.463; flora: mean = 7.206, SD = 7.414) as well as for the sub-ordinate categories (birds: mean = 7.289, SD = 7.499; mammals: mean = 7.347, SD = 7.582 ; fish: mean = 7.016, SD = 7.308; fruit: mean = 8.0, SD = 8.162; flowers: mean = 6.950, SD = 7.250; trees: mean = 7.206, SD = 7.414). Also, there were no differences between words that appear in the CELEX database and words that do not.

#### Procedure

Stimuli were presented in white upper case letters (maximum word size 9 × 3 cm) on a black background at a distance of 1.4 m using an LCD-projector situated outside the MEG chamber. Presentation time was 250 ms per word with inter-stimulus intervals varying between 1200 and 2000 ms and an average of 1600 ms.

Subjects were instructed to fixate a cross which was projected on the screen during stimulus-free intervals, to read each word and to respond by button press only to (rare) nouns depicting artificial objects. These 36 stimuli were interspersed with 10% probability in order to ensure sustained attention and processing involving/activating the mental lexicon. (Responses to these filler items were not analysed.)

### Data collection and analyses

Neuromagnetic signals were recorded continuously with a 148 channel whole head magnetometer (Magnes 2500 WH, 4D NeuroImaging Inc., San Diego) using a 0.1–100 Hz band-pass filter and sampled at a rate of 508 Hz. Additionally, vertical and horizontal EOG (electro-oculogram) were recorded for artifact control. Written informed consent was obtained from subjects prior to each MEG-session and the study was approved by the ethics committee of the University of Konstanz.

After external global noise subtraction continuous MEG and exclusion of artifact-contaminated epochs by visual inspection (EOG level > 100 μV, an MEG level > 5 pT, button press-induced artifacts), data were split into epochs of 900 ms length (including 100 ms pre-stimulus). This resulted on average in 50.2 MEG-traces per sub-category, subject and run (= 100.4 MEG-traces in sum per sub-category and subject). For every subject and for each of the six sub-ordinate categories, evoked magnetic fields (EMFs) were determined relative to a baseline of 100 ms pre-stimulus onset. For these average EMFs, cortical sources were determined using the minimum norm estimate (MNE, [31-33]), an objective inverse method to reconstruct the topography of the primary current underlying a magnetic field distribution ([34]). Cortical activity was approximated in a three-dimensional spherical source space of radius 10 cm fitted individually to the head-shape of the subjects (4-D Neuroimaging software). On this sphere 197 equidistant dipoles for the estimation of source activity were assumed. The source estimations (MNE) of the two runs per subcategory were averaged.

MNE amplitudes (in nAm) were averaged for 50 ms-segments across the 600-ms interval after stimulus onset and plotted separately for the two super- and six sub-ordinate categories, respectively. Since topographies for all six sub-categories were similar, time intervals and areas of interest for further analyses were determined for the average across all sub-categories. As apparent from Figure 1 foci of activity, that is, topographic peaks of MNE amplitudes, were obvious for three distinct areas in the left hemisphere. For these areas, the MNE amplitudes of seven dipoles were averaged for statistical analyses of stimulus effects. For each of the three areas (left-occipital, left-temporal and left-anterior), effects of categories on MNE-amplitudes were verified by two-way repeated measures Analyses of Variance comparing the three sub-ordinate categories each (as within-subject factor SUB-ORDINATE) within the two super-ordinate categories (as within-subject factor SUPER-ORDINATE) for successive 50-ms time intervals. Stars in figure 2 resemble significant main effects whereas stars in figure 3 resemble significant interactions. Newman-Keuls tests served for post-hoc explanation of interactions.

## Authors' contributions

RA designed the study, collected the data and accomplished the data analyses, and drafted the manuscript. BR was involved in planning and designing the study and substantially contributed to the manuscript.
